# Effect of a Spiritual Care Training Program to Build Knowledge, Competence, Confidence and Self-awareness Among Australian Health and Aged Care Staff: An Exploratory Study

**DOI:** 10.1007/s10943-023-01990-6

**Published:** 2024-01-11

**Authors:** Kate F. Jones, Matthew Kearney, Megan C. Best

**Affiliations:** 1 Institute for Ethics and Society, University of Notre Dame Australia, Broadway, PO Box 944, Sydney, NSW 2007 Australia; 2 St Vincent’s Hospital, Sydney, Australia

**Keywords:** Spirituality, Spiritual care, Healthcare, Aged care, Training

## Abstract

The aim of this study was to evaluate a new spiritual care training program with health and aged care staff. A four-module program was delivered to 44 participants at a large Catholic health and aged care provider in Australia. Pre, post and 6 week follow-up surveys were administered and included measures of spiritual care competency, confidence, perspectives of spirituality and spiritual care, spiritual well-being, and satisfaction. Paired sample *t*-tests showed total scores of participants’ spiritual well-being, spiritual care competency and confidence significantly improved following the training and were largely maintained at follow-up. Perspectives on spirituality and spiritual care did not significantly change over time.

## Introduction

The benefits of addressing spirituality in health and aged care are widely acknowledged in policy and research literature (Best et al., 2023; Egan et al., 2017; Jones et al., 2020b, 2021a; Meaningful Ageing Australia, 2016; Puchalski et al., 2014). A clear association between spiritual well-being and other positive health outcomes among those receiving care has been demonstrated (Best et al., 2016a, 2022b; Jones et al., 2018, 2022a). Furthermore, spiritual well-being has a protective role against burn-out and stress for those providing care (Bar-Sela et al., 2019; Chiang et al., 2021; De Diego-Cordero et al., 2022). Studies have shown that many of those entering health or aged care services wish to discuss their spiritual beliefs with healthcare staff (Best et al., 2014, 2015), and that staff believe spiritual care is the responsibility of all members of the multidisciplinary team when providing person-centred, holistic care (Jones et al., 2020b, 2022b). Although spiritual care is most often provided by spiritual care specialists (also known as pastoral care practitioners or chaplains) patients may want general staff to be aware of their spiritual needs (Best et al., 2015, 2022b). This may be a nurse, doctor, or member of the allied health or aged care team. Findings from recent studies suggest that such staff often do not feel equipped to provide spiritual care to the patients or residents they work with, (Best et al., 2016b, 2016c; Jones et al., 2020b; McSherry & Jamieson, 2011) but that provision increases with spiritual care training of staff (Jones et al., 2022b).

The interprofessional model of spiritual care (Balboni et al., 2014; Puchalski et al., 2014) proposes that all staff achieve a level of confidence to provide a general level of spiritual care, and refer on to specialist spiritual care as appropriate. A range of training programs have been developed internationally to assist health and aged care staff provide general spiritual care. A recent systematic review (Jones et al., 2021a) identified 55 evaluated programs. Most of these were developed in the USA and Europe and included a range of content and formats. Only two of the 55 programs were conducted in Australia, with a third recently conducted with rehabilitation professionals (Jones et al., 2020a, 2022b).

Studies of spiritual care with Australian healthcare professionals have revealed that perspectives on spirituality and spiritual care may differ substantially from countries where Christianity plays a culturally more significant role, such as the USA (Best et al., 2022a; Jones et al., 2021b). The latest census data reveal that more Australians are reporting “no religion” (38.9% in 2021, compared with 30.1% in 2016) and that those who identify as Christian are declining (43.9% in 2021 compared to 52.1% in 2016) (Australian Bureau of Statistics, 2022). Other faith groups are increasing in their proportion of the Australian population (since 2016, Hinduism has grown from 1.9 to 2.7%, and Islam has grown from 2.6 to 3.2%) (Australian Bureau of Statistics, 2017, Australian Bureau of Statistics, 2022). Furthermore, the spirituality of Aboriginal Australians is gaining greater visibility and acknowledgement, and the inclusion of the perspective of Aboriginal Australians in all healthcare training has been strongly recommended at government level (NSW Ministry of Health, 2020).

The aim of this study was to evaluate the pilot of a spiritual care training program (SCTP) developed by the authors with health and aged care staff from St Vincent’s Health Australia. The two main objectives of this study were to: (i) evaluate the feasibility and acceptability of the program for staff across a range of health and aged care settings; and (ii) investigate whether attending the program increased levels of self-rated knowledge, competency, and confidence in facilitating the spiritual well-being of healthcare patients and aged care residents.

## Methods

### Participants

Ethical approval for this study was obtained from Human Research Ethics Committees of St Vincent’s Hospital Sydney and the University of Notre Dame Australia (2021/ETH11141, 2021-129S). Eligible participants were healthcare or aged care staff working in participating hospitals or aged care facilities from St Vincent’s Health Australia. The participating facilities were: St Vincent’s Hospital Sydney, St Vincent’s Public Hospital Melbourne, and St Vincent’s Care Services (aged care). Managerial, administration, care and clinical staff were included.

### Study Procedure

Staff working in participating wards or facilities received an email from the pastoral care manager at their site, inviting them to participate in the study. The invitation included an online link to the participant information and consent form. Information was provided on what attending the training program and participating in the study would involve. If consent was provided by the participant, the participant proceeded to a survey which collected their demographic details and responses to items on spirituality and spiritual care.

### Development of Intervention

An empirically based training program was developed by the authors. Four studies were conducted to establish the content and format of the training (Best et al., 2022a; Jones et al., 2021a, 2021b, 2022c).

The first step taken was to conduct a systematic literature review of spiritual care training programs (Jones et al., 2021a). Facilitators of spiritual care training were identified and included: providing opportunities for reflection and practice, training being online, and the involvement of spiritual care specialists in training.

After the systematic review, the next step was to seek the expert opinion of staff working in health and aged care. Their views on the format and content of a program were obtained via a Delphi poll (Jones et al., 2021b). Preferred teaching methods included: the use of case studies; group discussion; incorporation of role-plays; self-directed learning; and reflection upon personal stories. Responses from participants highlighted the importance of training which incorporated screening for spiritual concerns and including a component which addressed one’s own spirituality and self-care.

The role of the spiritual care specialist on the team was emphasised in both the systematic review and the Delphi poll, and other studies (Geer et al., 2017a, 2017b) have demonstrated that their involvement in spiritual care training may be valuable for both staff and patients. To obtain the views of Australian spiritual care specialists on this matter, the authors conducted a focus group study (Jones et al., 2022c). Spiritual care specialists reported that they provided both informal and formal education to other staff about spiritual care. This included: building upon existing strengths and knowledge of staff; the use of stories and real-life examples; using language which made spirituality accessible; making training relevant and practical for staff; tapping into staff vocation; and building staff members’ awareness of their own spirituality.

Finally, an online trial of the Interprofessional Spiritual Care Education Curriculum (Puchalski et al., 2019), developed in the USA, was conducted. Although the training was effective in increasing levels of spiritual care competency and confidence among Australian healthcare staff, some of the context and associated processes were culturally unfamiliar. For example, spiritual history tools such as the Faith Importance Community Address (FICA) tool (Puchalski & Romer, 2000), which has been developed and used widely in spiritual care training in the USA, have not been well integrated into Australian healthcare (Jones et al., 2021b).

Each of these studies made a valuable contribution to the development of a program which would have relevance for Australians.

### Intervention Content

The training program consisted of four one-hour modules comprising: (1) an introduction to spirituality, (2) the use of screening questions, (3) an opportunity to reflect upon one’s own spiritual well-being, and (4) information on spiritual distress and how to manage this in patients or residents. The teaching objectives of each module are outlined in Table 1.

**Table 1:** Teaching objectives

Module	Objectives
1	• To increase staff awareness of the importance of spirituality to patient/resident well-being, and the different sources of spiritual strength that patients or residents may draw upon
2	• To increase staff knowledge, skill and confidence in spiritual screening, recognising cues and documenting spiritual needs • To increase staff communication skills in listening and person-centred care • To increase staff awareness of the pastoral care role and how to refer
3	• To build upon participants’ awareness of the need for self-care • To assist staff to identify sources of spiritual strength or connectedness that are meaningful in their own lives • To enhance participants’ ability to self-reflect
4	• To increase staff knowledge, skill and confidence in spiritual screening and recognising cues for spiritual distress • To increase staff communication skills in listening and providing a compassionate presence • To increase staff awareness of pastoral care support and other supports where needed

There was flexibility for the program to be run on-line or in-person, and an abbreviated program was made available for those services which could only commit to four 30 min modules. One-day programs were also possible but not used during the pilot. Teaching methods included didactic input, group discussion, personal reflection, and case studies employing written or video content. The perspectives of Aboriginal Australians were integrated throughout the program through video content and suggested readings. All sessions were facilitated by pastoral care managers or pastoral care practitioners who were spiritual care specialists who had received training in facilitating the program material.

### Data Collection

Surveys were administered at three time points: *T*0 (baseline): up to 2 weeks before attending the first module, *T*1: at the completion of the fourth module, and *T*2: 6 weeks after completion of the program. All surveys were completed online.

The following information was collected at *T*0 only.

#### Demographic Data

Details of participants’ age category, gender, years of work experience, self-rated spirituality and religiosity, religious affiliation and ethnicity or ancestry were collected. Participants were also asked if they had ever received formal training or education in spiritual care.

The following measures were administered at all three time points (*T*0, *T*1, *T*2).

#### Staff Perceptions on Spirituality

The Spirituality and Spiritual Care Rating Scale (SSCRS) (McSherry et al., 2002) is a validated 17-item measure of spirituality and spiritual care and uses a five-point scale ranging from 1 “strongly disagree” to 5 “strongly agree”. A four factor model of the SSCRS (Ross et al., 2014) was used in this study: Existential Spirituality (the view that spirituality is concerned with people’s sense of meaning, purpose, value, peace, and creativity; 5 items); Religiosity (the view that spirituality is only about religious beliefs; 3 items); Spiritual Care (the view of spiritual care in its broadest sense including religious and existential elements, for example facilitating religious rituals and showing kindness; 5 items); and Personal Care (taking account of people’s beliefs, values and dignity; 3 items), with one item contributing to the score of two of the sub-scales. As the factors focus on different aspects of spirituality and spiritual care, and because one of the factors (Religiosity) may decrease rather than increase over time (Jones et al., 2020a) (and therefore impact upon the total score), in this study we have only reported the sub-scale scores. Scores are calculated by averaging the mean for the relevant items (all scores therefore range from 1to 5) (Ross et al., 2014). The SSCRS has a modest level of internal consistency (Cronbach’s alpha = 0.64) (McSherry et al., 2002) and has been used in a range of health settings in previous Australian studies (Austin et al., 2016; Jones et al., 2020a).

#### Self-rated Spiritual Care Competency and Confidence

The Spiritual Care Competency Scale (SCCS) (van Leeuwen et al., 2009) is a validated 27-item measure designed to assess nurses’ competencies in providing spiritual care. It collects self-reported ratings of competency in relation to six domains: (i) assessment and implementation of spiritual care; (ii) professionalisation and improving the quality of spiritual care; (iii) personal support and patient counselling; (iv) referral to professionals; (v) attitude towards the patient’s spirituality; and (vi) communication. Responses are given by five-point Likert scales, from ‘completely disagree’ to ‘completely agree,’ with total possible scores ranging from 27 to 135. Staff confidence levels regarding spiritual care were rated according to the six SCCS domains, on a scale from 0 to 10, with possible total scores ranging from 0 to 60. Higher scores indicated higher levels of confidence. As the SCCS was originally designed for nursing staff, minor adjustments were made to the wording to ensure suitability for other staff. The SCCS has been successfully administered in previous Australian studies (Jones et al., 2020a).

#### Spiritual Wellbeing

The long-form World Health Organization’s Quality of Life instrument contains a spiritual, religious, and personal beliefs domain (WHOQOL-SRPB) comprising 132 items. This validated measure has been tested cross culturally across 18 different cultures. A brief nine-item version of this domain was developed and tested by Skevington et al. (2013) and was used in this study to measure participants’ spiritual well-being. Items are rated on a five-point Likert scale (Not at all, A little, A moderate amount, Very much, An extreme amount), with participants indicating the extent to which they are assisted by various sources of spiritual strength and well-being.

The following data were only collected at *T*1 and *T*2.

#### Satisfaction with Program

At *T*1, participants were invited to rate their satisfaction with the program on a range of aspects using a five-point Likert scale. They were also provided with an opportunity to enter free text responses regarding things they liked about the program, things that could be improved, and something they hoped to do differently in the future in their own spiritual care practice. At *T*2, participants were asked to reflect upon anything they were doing differently as a result of attending the program.

### Data Analysis

An exploratory statistical analysis was conducted due to the small sample size and high attrition rate during data collection. The analysis was conducted using IBM Statistics Version 26. Descriptive statistics were employed to analyse the demographic data and responses to the questionnaires. Data were inspected for normality (Kolmogorov–Smirnov test). As the majority of variables achieved normality, a paired sample *t*-test was used to measure differences relating to competency and confidence at each timepoint. Cohen’s d was used a measure of effect size.

Some qualitative data were collected from participants via open-ended questions in the survey. These responses were analysed for themes, using a content analysis.

## Results

A total of 64 participants enrolled in the study and provided consent. Of these 44 proceeded with the intervention (see Table 2 for demographic details). A large proportion of the participants were female, holding managerial or administration positions, and born in Australia. Just over half the participants identified as Christian or Catholic, with over a quarter of participants strongly agreeing that they were “spiritual.” A total of seven group programs were held between May 2022 and January 2023. The training was run in a range of settings, including aged care, online managerial meetings, and on hospital wards, using a range of formats including online and in-person, and full as well as abbreviated versions (see Table 3).

**Table 2:** Participant demographic characteristics (*n* = 44)

Variable		*N* (%)
Age group	20 or below	1 (2.3)
	21–29	1 (2.3)
	30–39	4 (9.1)
	40–49	13 (29.5)
	50–59	17 (38.6)
	60 or above	8 (18.2)
Sex	Female	38 (86.4)
	Male	6 (13.6)
Ethnicity	Australian	29 (65.9)
	European/English	5 (11.4)
	Asian	4 (9.1)
	African	3 (6.8)
	North American	1 (2.3)
	South American	1 (2.3)
	Torres Strait Islander/Asian	1 (2.3)
I am a religious person	Strongly disagree	2 (4.5)
	Disagree	7 (15.9)
	Neither agree nor disagree	14 (31.8)
	Agree	15 (34.1)
	Strongly agree	6 (13.6)
I am a spiritual person	Strongly disagree	1 (2.3)
	Disagree	1 (2.3)
	Neither agree nor disagree	7 (15.9)
	Agree	24 (54.5)
	Strongly agree	12 (27.3)
Religious affiliation	Christian	23 (52.3)
	Jewish	2 (4.5)
	None	19 (43.2)
Previous formal spiritual care training	Yes	7 (15.9)
	No	37 (84.1)
Discipline	Manager (aged care)	20 (45.5)
	Assistant in nursing	5 (11.3)
	Nursing	7 (15.9)
	Social worker	2 (4.5)
	Psychologist	1 (2.3)
	Administration/management	5 (11.4)
	Education/quality	3 (6.8)
	Not reported	1 (2.3)
Years work experience *M*(SD)		16.7 (10.9)

**Table 3:** Group format

Group number	Context	Number of attendees	Online (O)/in-person (I)	Full (F)/abbreviated (A)
1	Health	4	I	F
2	Aged care	10	I	A
3	Health	8	I	A
4	Aged care	8	O	A
5	Aged care	10	O	A
6	Aged care	2	O	A
7	Health	3	O	F

Of the total group of participants, 100% completed surveys at *T*0, 68% at *T*1 and 36% at *T*2. Paired sample *t*-tests showed total scores of spiritual well-being, spiritual care competency and confidence significantly and immediately improved following the training (*T*O–*T*1). These improvements were largely maintained at follow-up (see Table 4). Effect sizes were medium, ranging from 0.43 to 0.66. Perspectives on spirituality and spiritual care (SSCRS) did not significantly change from *T*0 to *T*1, however there were some small improvements between *T*0 and *T*2 on the two SSCRS factors of Spiritual Care and Existential Care (effect sizes were medium, ranging from 0.58 to 0.62).

**Table 4:** Changes to spiritual care perspectives, competency, confidence, and spiritual well-being over time

	Pre-program (*n* = 44) *M*(SD)	Post-program (*n* = 30) *M*(SD)	*t*	*p*
Perspectives—existential care	4.01 (0.51)	4.11 (0.53)	− 1.1	0.295
Perspectives—religiosity	1.87 (0.67)	1.77 (0.9)	− 0.6	0.553
Perspectives—spiritual care	4.36 (0.44)	4.48 (0.52)	− 1.3	0.196
Perspectives—personal care	3.9 (0.5)	3.97 (0.63)	− 0.7	0.519
Competency	102.5 (18.8)	109.7 (14.1)	− 2.4	0.024^a^
Confidence	41.5 (13.9)	47.3 (9.2)	− 3.1	0.005^b^
Spiritual well-being	33.4 (5.8)	35.0 (5.7)	− 2.6	0.015^a^

	Pre (*n* = 44) *M*(SD)	Follow-up (*n* = 17) *M*(SD)	*t*	*p*
Perspectives—existential care	3.88 (0.44)	4.18 (0.46)	− 2.4	0.03^a^
Perspectives—religiosity	1.67 (0.5)	1.50 (0.7)	1.07	0.299
Perspectives—spiritual Care	4.26 (0.36)	4.50 (0.36)	− 2.6	0.021^a^
Perspectives—personal Care	3.90 (0.37)	4.07 (0.43)	− 1.9	0.076
Competency	100.8 (16.7)	110.0 (10.0)	2.7	0.016^a^
Confidence	41.7 (13.3)	47.9 (7.8)	− 2.3	0.038^a^
Spiritual well-being	33.6 (6.1)	35.7 (6.4)	− 2.0	0.06

^a^*p* < 0.05

^b^*p* < 0.01

The satisfaction data indicated that over three-quarters of the participants were very or extremely satisfied with the overall program (see Table 5). The open-ended responses collected at *T*1 and *T*2 revealed that participants enjoyed the videos, the flexibility of the facilitators when running the sessions, time to interact with others, and an opportunity to reflect upon their own spirituality. Aspects of the course they thought could be improved included: allocating more time for reflection and discussion and providing pre-group reading to shorten the session duration. When asked at *T*2 what they might do differently as a result of attending the program, responses included: paying more attention to little things and listening more carefully to patients or residents, asking patients/residents more questions about spirituality, creating opportunities for improving spiritual outcomes of patients or residents, holding open discussions about spirituality with team members, and being more aware of the role of pastoral care. Although much of the training was conducted online (due to the COVID-19 pandemic), when it was held in person participants commented on how receiving care for themselves from the pastoral care facilitators, such as a cup of tea and a biscuit, were valued.

**Table 5:** Satisfaction with the program (*n* = 32**)**

	Very unsatisfied *n* (%)	Unsatisfied *n* (%)	Moderately satisfied *n* (%)	Very satisfied *n* (%)	Extremely satisfied *n* (%)
Time allocated for each module	1 (3.1)	2 (6.3)	9 (28.1)	17 (53.1)	3 (9.4)
Balance between theoretical/practice	0 (0)	3 (9.4)	9 (28.1)	18 (56.3)	2 (6.3)
Program content	0 (0)	0 (0)	9 (28.1)	18 (56.3)	5 (15.6)
Exercises	0 (0)	0 (0)	9 (28.1)	20 (62.5)	3 (9.4)
Level of interaction encouraged	0 (0)	2 (6.3)	8 (56.3)	14 ()	8 (17.8)
Relevant language and case examples	0 (0)	0 (0)	9 (28.1)	18 (45.0)	5 (15.6)
Overall program	0 (0)	0 (0)	7 (21.8)	21 (65.6)	4 (12.5)

## Discussion

This was the first study we know of in Australia to evaluate a spiritual care training program across a range of health and aged care settings. Satisfaction with the program was generally high, and increases in staff levels of spiritual care competency, confidence and spiritual well-being were observed and maintained over time. There were challenges experienced in trialling the program during the COVID-19 pandemic and this had an impact upon recruitment and retention rates as well as influencing the way the content was delivered at some sites.

Increases in spiritual care competency and confidence have been observed after other spiritual care training interventions (Best et al., 2022a; Jones et al., 2020a; Ross et al., 2014). Similarities can be identified between this study and an Australian study by Jones et al. (2020a), which was conducted with rehabilitation professionals. Similar to that study, there were mixed findings regarding the impact of training on participant perspective*s* regarding spirituality and spiritual care, measured by the SSCRS. In both studies it was observed that the SSCRS ‘Religiosity’ sub-scale performed differently to the other SSCRS sub-scales. Higher scores on the ‘Religiosity’ sub-scale are indicative of a viewpoint that spirituality is only about religious beliefs. As the training program introduced participants to a broad conceptualisation of spirituality, a slight (non-significant) downward trend in these scores over time was not surprising. There was also no significant change in participant perspectives on the ‘Personal Care’ sub-scale, a finding also observed in Jones et al. (2020a). As noted by those authors, most health and aged care staff are well versed in person-centred care, and already have a good understanding of this component of spiritual care so minimal change is not unexpected. There were however significant changes at follow-up on both the ‘Spiritual Care’ and ‘Existential Care’ sub-scales, suggesting that staff may benefit from reflecting upon these aspects of spirituality over time and with patient contact. However, numbers at *T*2 were much smaller than *T*1 so it is difficult to draw strong conclusions. Compared with Jones et al. (2020a), participant levels at T0 on both spiritual care competency and confidence were higher in this study, possibly explained by the Catholic healthcare context within which this study was conducted where there is greater exposure to pastoral care and support. Further research is needed to measure the impact of staff training on the experience of the patient or resident.

In addition to changes in participant levels of spiritual care competency, confidence and knowledge, this study also examined changes in participant levels of spiritual well-being. Spiritual well-being levels increased from *T*0 to *T*1, levels which appeared to be largely sustained at *T*2. The benefits of spiritual well-being for healthcare staff such as nurses and medical students have been known for some time (De Diego-Cordero et al., 2022; Wachholtz & Rogoff, 2013). In a recent scoping review, De Diego-Cordero et al. (2022) found that spirituality was correlated with lower levels of burn-out, exhaustion and depersonalisation among nursing staff. Likewise, spiritual well-being has been associated with higher levels of satisfaction with life for medical students, and lower likelihood of burn-out and psychological distress (Wachholtz & Rogoff, 2013). The importance of these findings for health and aged care since the COVID-19 pandemic has increased, with burn-out rates reported to be as high as 49–58% in some healthcare settings at the height of the pandemic (Dobson et al., 2021; Gualano et al., 2021).

COVID-19 undoubtedly had an impact on staff recruitment and retention rates throughout this study due to lockdowns, social distancing, and staff illness. However, there were some benefits gained, such as the access to and familiarity of participants with online conferencing software, enabling training to be conducted even when strict social distancing guidelines were in place. The benefits of online training in the area of spiritual care have been noted by others (Pearce et al., 2020), and in an increasingly pressurised health/aged care context may increase uptake for busy staff. Flexibility was appreciated by participants with busy schedules, and the abbreviated version of the program was often chosen over a full program due to staff workloads. This increased the number of participants who could complete the training. However, several participants would have preferred more time to reflect upon and discuss the material, suggesting that a need for staff to be able to do so should also be considered. Longer periods between sessions may meet this need and should be trialled in future studies.

Previous studies have identified that the involvement of spiritual care specialists is a facilitator for spiritual care training (Jones et al., 2021a). Such involvement increases awareness of their role, models how spiritual care can be administered, and builds a relationship between staff and those providing specialist care (Jones et al., 2022c). This was found to be the case in this study, with several participants commenting on how they were more aware of the role of spiritual care specialists following training and enjoyed both receiving and learning about spiritual care.

### Limitations

There were several limitations of this study. As mentioned above, the COVID-19 pandemic impacted recruitment and the sample size was small. COVID-19 outbreaks sometimes delayed the running of groups in aged care facilities, and attrition occurred as a result of staff sickness or quarantine. The predominance of females, older staff, and a lack of ethnic or religious diversity among participants resulted in a largely homogenous sample. As St Vincent’s Health Australia is a Catholic healthcare provider, participants may have had a stronger affiliation with Christian values and beliefs than others in a more secular setting.

## Conclusion

This study has demonstrated that spiritual care training is well received by health and aged staff when presented in a format which is flexible to their needs. Spiritual care training increased levels of spiritual care competency, confidence and levels of staff spiritual well-being which may play a protective role against burn-out. As very few spiritual care training programs have been developed and conducted in Australia, there is potential to further enhance the role of such education in Australian health and aged care settings. More research in this area will provide additional information about how the spiritual needs and strengths of patients, residents and staff can be better identified and addressed by health and aged care facilities leading to better outcomes for all.
